# Efficacy of Yun-Type Optimized Pelvic Floor Training Therapy for Middle-Aged Women With Severe Overactive Bladder: A Randomized Clinical Trial

**DOI:** 10.3389/fsurg.2021.670123

**Published:** 2021-07-14

**Authors:** Chaoliang Shi, Dan Zhou, Wandong Yu, Wei Jiao, Guowei Shi, Yangyun Wang

**Affiliations:** ^1^Department of Urology, The Fifth People's Hospital of Shanghai, Fudan University, Shanghai, China; ^2^School of Health and Social Care, Shanghai Urban Construction Vocational College, Shanghai, China

**Keywords:** overactive bladder, yun-type pelvic floor optimal training, pelvic floor muscle training, drug therapy, randomized clinical trial

## Abstract

**Background:** This study aimed to evaluate the clinical efficacy of Yun-type optimized pelvic floor training therapy for middle-aged women with severe overactive bladder (OAB).

**Methods:** This randomized, observer-blinded, parallel-group controlled clinical trial included 108 middle-age women with severe OAB and assigned them to the intervention group (treated with combination of Yun-type optimized pelvic floor training with solifenacin for 12 weeks) and control group (treated with solifenacin for 6 weeks and, after 2 weeks of elution, received the combination of Yun-type optimized pelvic floor training and solifenacin for 6 weeks). The outcomes associated with OAB, pelvic floor muscle (PFM) function, and sexual function were compared after 6 and 12/14 weeks of treatment.

**Results:** The primary variables were OAB-associated outcomes, including overactive bladder symptom score (OABSS), urgent urination, urine, nocturia, urge urinary incontinence, patient's perception of bladder condition, urogenital distress inventory-6, incontinence impact questionnaire-7, voiding volume, average flow rate, and maximum flow rate. The secondary variables were indicators related to PFM function and sexual function. These indicators were significantly improved in both groups after interventions. Notably, the improvements in most of these indicators were superior in the intervention group than in the control group after 6 weeks and 12/14 weeks of treatment.

**Conclusions:** The use of Yun-type optimized pelvic floor training adds to the benefits of solifenacin regarding severe OAB-associated outcomes, PMF function, and sexual function in middle-aged women with severe OAB. Combining Yun-type optimized pelvic floor training with traditional drug therapies may improve clinical outcomes in patients with severe OAB.

**Trial Registration:** ChiCTR-INR-17012189.

## Background

Overactive bladder (OAB) is a chronic condition with a negative impact on quality of life (QoL) (1, 2). It is characterized by urinary urgency with or without urgency urinary incontinence (UUI), usually accompanied by urinary frequency and nocturia (3). The prevalence of OAB in the general population is estimated to be 14–16% (4) and increases with age; ~30% of women over 65 years old are diagnosed with OAB (5). The symptoms of OAB have adverse impacts on sleep, mental health, work productivity, and QoL (6, 7). OAB is also associated with lower sexual desire, sexual arousal, orgasm, and sexual satisfaction, resulting in female sexual dysfunction (SD) (6). Moreover, OAB imposes a significant economic burden surpassing $14 billion with societal costs for older women (8). Therefore, it is important to develop an effective treatment strategy for management of OAB.

In clinical practice, OAB is usually treated with antimuscarinic agents such as solifenacin, tolterodine, fesoterodine, trospium, darifenacin, and propantheline to inhibit detrusor contractions (9, 10). However, these antimuscarinic agents have some side effects such as headache, nausea, dry eyes and mouth, and constipation (11). The International Continence Society (ICS) committee has suggested that the cause of OAB is from the detrusor itself. Stress and urge symptoms may both derive from the same anatomical defect, a lax vagina. This laxity may be caused by defects within the vaginal wall itself, or its supporting structures i.e., ligaments, muscles, and their connective tissue insertions, which may dissipate the muscle contraction, causing stress incontinence, and/or activation of an inappropriate micturition reflex, leading to symptoms of frequency, urgency, nocturia with or without urine loss (12–14). Pelvic floor muscle (PFM) training (PFMT) has been shown to reduce detrusor activity and improve QoL in patients with OAB (15) and has been recommended as first-line therapy for women with stress and other types of urinary incontinence (16). PFMT involves the contraction of the puborectal anal sphincter and external urethral muscles that inhibits the detrusor muscle contraction, justifying its use in OAB treatment (17). However, PFMT is monotonous, leading to poor compliance and lack of improvement of OAB symptoms (18). Despite the developments in behavioral medicine, it is still unclear whether making PFMT fashionable and interesting has a better clinical effect on severe OAB than traditional PFMT.

In this study, we established a new safe and effective groin-pelvic floor functional reconstruction training method called Yun-type optimized pelvic floor training. This method combines PFMT with fashionable and sexy dance moves. We then aimed to assess the efficacy and safety of this method for patients with severe OAB using a randomized, observer-blinded, parallel-group controlled clinical trial. Our results will provide new insights for optimizing the treatment of OAB.

## Methods

### Study Design and Patients

This study was designed as a randomized, observer-blinded, parallel-group controlled clinical trial. Written informed consent was obtained from all participants, and the protocol was reviewed and approved by the Ethics Committee of the Shanghai Fifth People's Hospital Affiliated to Fudan University (No. 2017-041). The clinical trial registration was ChiCTR-INR-17012189.

A total of 108 women with severe OAB were recruited from Shanghai Fifth People's Hospital, Fudan University. The inclusion criteria were listed as follows: (1) married women aged 30–45 years; nullipara or gave birth by cesarean section; (2) urgent urination with or without UUI, able to complete a 3-day urination diary, ≥8 episodes of urgent urination within 24 h over 3 days, and ≥2 episodes of urination at night, with urine volume <200 ml each time; (3) the total score of overactive symptom bladder score (OABSS) ≥ 12 points and urgent urination score ≥ 2 points; (4) the female sexual function index (FSFI) <25 points; (5) stable sexual partners; (6) duration of disease > 3 months; and (7) no drug use during the 3 months before selection. The exclusion criteria were as follows: (1) patients had no sexual experience, were breastfeeding or in menopause, or had pelvic floor organ prolapse, gynecological inflammation, tumor, and other endocrine diseases; (2) patients with stress incontinence or mixed urinary incontinence dominated by stress urinary incontinence; (3) patients had urinary infection or chronic inflammation such as interstitial cystitis, bladder stones, and malignant tumors; (4) patients had a history of transurethral and pelvic surgery; (5) patients had diabetes, glaucoma, myasthenia gravis, ulcerative colitis, severe constipation, and dyspepsia; (6) post-voiding residual (PVR) > 50 ml; (7) patients had used M3 receptor blockers or other drugs to treat OAB; (8) patients were intolerant to the adverse effects of solifenacin; and (9) patients were unable to complete treatment course, or could not complete the Yun-type optimized pelvic floor training, urination diary, blood tests, and urodynamic tests.

### Interventions

The patients were randomly assigned into intervention group and control group. Randomization was performed in a double-blinded manner through a central computerized system by using randomly permuted blocks (size = 6). Specifically, 6 community health service centers in our hospital and the same area were grouped using block randomization, and each group included 18 cases. The case was diagnosed in our hospital and transferred to the corresponding community for treatment. Patients in the intervention group received Yun-type optimized pelvic floor training therapy combined with solifenacin (10 mg/d) for 12 weeks. Patients in the control group were first treated with solifenacin (10 mg/d) for 6 weeks and, after 2 weeks of elution, received a combination of Yun-type optimized pelvic floor training therapy and solifenacin (10 mg/d) for 6 weeks. The details regarding the Yun-type optimized pelvic floor training therapy (19–21) were as follows: each patient wore a 488 bi metal waist chain with a weight of (0.8+0.1) kg at the waist and hip during training. The training included 15 min of crotch base warm-up exercises; 25 min of crotch shaking, crotch twisting, crotch shaking, crotch sending, upper crotch, lower crotch, and sago shaking under the condition of contracting the anus and vagina (the specific time and rhythm of anus contraction and vagina contraction were conducted according to the command of the head coach); and the last 15 min of PFM strengthening and body relaxation exercises with soothing music: in detail, legs flexed and slightly apart, followed by slow frequency (contraction of the anus and vagina lasts for 30 s during inhalation, and relaxation lasts for 10 s during exhalation) and fast frequency (contraction of the anus and vagina for 2 s during inhaling, and relaxation for 2 s during exhalation) courses, 45 times per course. Yun-type optimized pelvic floor training was done at least four times a week.

### Endpoints

The primary endpoints were OAB-related outcomes such as OABSS, urgent urination, urine, nocturia, UUI, patient's perception of bladder condition (PPBC), urogenital distress inventory-6 (UDI-6), incontinence impact questionnaire-7 (IIQ-7), voiding volume (VV), average flow rate (Qave), maximum flow rate (Qmax), and postvoid residual urine volume (PVR)]. The secondary endpoints were outcomes related to PFM function such as type I/II muscle strength and PFM tension as well as sexual function-associated outcomes such as FSFI, sexual desire, arousal, damp, orgasm, pain, and satisfaction. The PFM assessment was measured using the Glazer Protocol as previous described (22). These outcomes were measured before treatment and 6 weeks and 12/14 weeks after.

### Statistical Analysis

Continuous data were tested for normality. Normally distributed data were expressed as mean ± standard deviation and differences between groups were analyzed with the *t*-test, whereas data that were not normally distributed were presented as median (interquartile range) and compared using the Mann–Whitney U test. Qualitative data were represented as n (%) and analyzed by chi-square (χ2) test. The repeated measures ANOVA was used to analyze the main effect of study group, the main effect of time and the interaction between group and time on the changes in endpoints, including age as a covariate. The required sample size was calculated to have 90% power to detect a difference of endpoints the intervention group and to have 60% power in the control group using unpaired Mann–Whitney *U*-test. The cutoff value was set as 0.1, α = 0.05, 1-β = 0.8, and the sample size was 1:1. As result, the sample size should be more 102 cases, 51 cases in each group. Assuming that no more than 3 cases were lost to follow-up, thus, 108 cases were enrolled in total. All statistical data were processed with the Statistical Package for the Social Sciences (SPSS) software version 19.0 (SPSS Inc., Chicago, IL, USA). A two-sided *P*-value < 0.05 was considered statistically significant.

## Results

### Baseline Characteristics of Patients

A total of 108 patients with severe OAB were randomly divided into the intervention group (*n* = 53) and control group (*n* = 55). The baseline characteristics of patients are shown in Table 1. There were no significant differences between the two groups regarding age (*P* = 0.5603), OABSS (*P* = 0.1831), urgent urination (*P* = 0.3319), urine (*P* = 0.1079), nocturia (*P* = 0.5442), UUI (*P* = 0.8844), PPBC (*P* = 0.0864), UDI-6 (*P* = 0.2872), IIQ-7 (*P* = 0.6820), PVR (*P* = 0.5442), type I muscle strength (*P* = 0.3733), type II muscle strength (*P* = 0.5787), FSFI (*P* = 0.6645), sexual desire (*P* = 0.1972), arousal (*P* = 0.6450), damp (*P* = 0.6135), orgasm (*P* = 0.7858), pain (*P* = 0.1357), and satisfaction (*P* = 0.0745). However, significant differences were obtained between the two groups regarding VV (*P* = 0.7858), Qave (*P* < 0.0001), Qmax (*P* = 0.0101), and PFM tension (*P* = 0.0197).

**Table 1:** The baseline characteristics of patients.

**Characteristics**	**Control** ** (*n* = 55)**	**Intervention** ** (*n* = 53)**	***P***
Age	37.00 (32.00, 42.00)	35.00 (33.00, 42.00)	0.5603
OABSS	12.00 (12.00, 12.00)	12.00 (12.00, 13.00)	0.1831
Urine			0.1079
1	14 (25.45)	7 (13.21)	
2	41 (74.55)	46 (86.79)	
Nocturia			0.5442
0	1 (1.82)	0 (0.00)	
2	12 (21.82)	14 (26.42)	
3	42 (76.36)	39 (73.58)	
Urgent urination			0.3319
4	29 (52.73)	23 (43.40)	
5	26 (47.27)	30 (56.60)	
UUI			0.8844
2	5 (9.09)	6 (11.32)	
3	27 (49.09)	25 (47.17)	
4	18 (32.73)	19 (35.85)	
5	5 (9.09)	3 (5.66)	
PPBC			0.0864
3	0 (0.00)	6 (11.32)	
4	11 (20.00)	9 (16.98)	
5	23 (41.82)	19 (35.85)	
6	21 (38.18)	19 (35.85)	
UDI-6	45.00 (35.00, 55.00)	45.00 (35.00, 55.00)	0.2872
IIQ-7	60.00 (55.00, 70.00)	60.00 (55.00, 65.00)	0.6820
VV	190.00 (175.00, 200.00)	8.00 (6.00, 9.00)	<0.0001
Qave	8.00 (6.00, 8.00)	190.00 (180.00, 200.00)	<0.0001
Qmax	19.00 (16.00, 21.00)	20.00 (18.00, 23.00)	0.0101
PVR	14.00 (8.00, 25.00)	15.00 (8.00, 20.00)	0.4996
Type I muscle strength	16.48 (1.87)	16.12 (2.25)	0.3733
Type II muscle strength	22.82 (1.92)	22.60 (2.11)	0.5787
Pelvic floor muscle tension	11.19 (2.25)	12.18 (2.09)	0.0197
FSFI	21.20 (19.20, 22.40)	21.20 (19.00, 22.40)	0.6645
Sexual desire	3.00 (2.40, 3.00)	2.40 (2.40, 3.00)	0.1972
Arousal	3.00 (2.40, 3.00)	3.00 (2.40, 3.00)	0.6450
Damp	3.60 (3.60, 4.20)	3.60 (3.60, 4.20)	0.6135
Climax	3.60 (2.80,3.60)	3.60 (3.20,3.60)	0.7858
Pain	4.00 (3.60, 4.40)	4.00 (4.00, 4.40)	0.1357
Satisfaction	3.80 (3.60, 4.40)	4.40 (3.60, 4.80)	0.0745

### Outcome Analyses

The primary endpoints were OAB-associated outcomes. The results showed that OABSS, urgent urination, urine, nocturia, UUI, PPBC, UDI-6, and IIQ-7 were significantly decreased after treatment in both groups compared with before treatment, whereas VV, Qave, and Qmax were significantly increased in both groups (Table 2). Notably, the improvements in these indicators in the intervention group were superior to the control group after 6 weeks of treatment. However, apart from OABSS, nocturia, urgent urination, PPBC, VV, Qave, and Qmax, other indicators were not significantly different between groups after 12/14 weeks of treatment (Table 2). There were no significant differences in PVR levels between the two groups after both 6 and 12/14 weeks of treatment (Table 2).

**Table 2:** Outcomes associated with overactive bladder symptom.

**Outcomes**	**Groups**	**Before intervention**	**6 weeks**	**12 weeks**	**Time effect**	**Group effect**	**Time × Group effect**
OABSS	Control group	12.35 (0.75)	10.24 (1.12)	4.98 (1.66)	*F* = 35.08	*F* = 92.21	*F* = 232.15
	Intervention group	12.53 (0.89)	4.94 (1.75)^***^	4.06 (1.60)^**^	<0.0001	<0.0001	<0.0001
Urine	Control group	1.75 (0.44)	1.44 (0.69)	0.53 (0.50)	*F* = 13.79	*F* = 13.00	*F* = 34.42
	Intervention group	1.87 (0.34)	0.64 (0.62)^***^	0.45 (0.50)	<0.0001	0.0005	<0.0001
Nocturia	Control group	2.73 (0.56)	2.47 (0.54)	1.13 (0.64)	*F* = 5.85	*F* = 43.68	*F* = 66.02
	Intervention group	2.74 (0.45)	1.09 (0.63)^***^	0.89(0.58)^*^	0.0053	<0.0001	<0.0001
Urgent urination	Control group	4.47 (0.50)	4.05 (0.62)	2.27 (0.56)	*F* = 4.21	*F* = 65.74	*F* = 112.70
	Intervention group	4.57 (0.50)	2.15 (0.74)^***^	1.91 (0.77)^**^	0.0162	<0.0001	<0.0001
UUI	Control group	3.42 (0.79)	2.27 (0.78)	1.05 (0.78)	*F* = 24.20	*F* = 15.91	*F* = 52.44
	Intervention group	3.36 (0.76)	1.04 (0.73)^***^	0.81 (0.68)	<0.0001	0.0001	<0.0001
PPBC	Control group	5.18 (0.75)	3.13 (0.84)	2.09 (0.55)	*F* = 18.90	*F* = 19.96	*F* = 17.71
	Intervention group	4.96 (1.00)	2.08 (0.70)^***^	1.85 (0.57)^*^	<0.0001	<0.0001	<0.0001
UDI-6	Control group	46.00 (10.06)	27.67 (10.55)	16.91 (8.09)	*F* = 11.13	*F* = 12.31	*F* = 14.03
	Intervention group	44.21 (9.65)	16.94 (7.56)^***^	15.36 (7.31)	<0.0001	0.0007	<0.0001
IIQ-7	Control group	62.07 (10.10)	26.00 (10.59)	15.76 (7.60)	*F* = 8.20	*F* = 2.13	*F* = 8.74
	Intervention group	61.77 (9.65)	19.15 (8.87)^***^	17.30 (7.56)	0.0004	0.1478	0.0002
VV	Control group	189.36 (23.53)	228.91 (31.49)	268.55 (34.89)	*F* = 4.88	*F* = 3,838.08	*F* = 192.66
	Intervention group	7.66 (1.44)^***^	9.67 (2.19)^***^	10.29 (2.65)^***^	0.0127	<0.0001	<0.0001
Qave	Control group	7.71 (1.40)	8.58 (1.86)	10.25 (1.84)	*F* = 0.04	*F* = 3,915.31	*F* = 268.67
	Intervention group	189.25 (23.70)^***^	270.85 (39.44)^***^	276.79 (38.00)^***^	0.9608	<0.0001	<0.0001
Qmax	Control group	18.87 (3.14)	20.51 (3.97)	24.15 (1.94)	*F* = 0.91	*F* = 20.75	*F* = 9.41
	Intervention group	20.53 (3.18)^**^	24.08 (3.06)^***^	25.19 (2.59)^*^	0.4010	<0.0001	0.0001
PVR	Control group	14.78 (9.38)	14.98 (8.06)	14.69 (8.21)	*F* = 0.06	*F* = 0.14	*F* = 0.03
	Intervention group	15.17 (8.20)	15.57 (6.40)	15.21 (6.69)	0.9393	0.7109	0.9690

* indicated the comparison between groups within each time period, *P < 0.05,

** *P < 0.01, and*

*** *P < 0.001*.

The secondary endpoints were indicators related to PFM function and sexual function. In terms of indicators related to PFM function, we found that type I and type II muscle strengths in both groups were dramatically enhanced after treatment compared to those before treatment, while the PFM tension was significantly weakened (Table 3). Moreover, the improvements in these indicators in the intervention group were all superior to the control group after 6 and 12/14 weeks of treatment (Table 3). In terms of sexual function-associated outcomes, the results revealed that patients in both groups had significant improvements in FSFI, sexual desire, arousal, orgasm, and satisfaction after treatment compared to those before treatment (Table 4). Additionally, the improvement of these indicators was significantly larger in the intervention group than in the control group after 6 weeks of treatment, whereas no significant differences were noted between the groups after 12/14 weeks of treatment (Table 4). However, there was no remarkable difference in vaginal damp and sexual pain after treatment in both groups (Table 4).

**Table 3:** Outcomes associated with pelvic floor muscle function.

**Outcomes**	**Groups**	**Before intervention**	**6 weeks**	**12 weeks**	**Time effect**	**Group effect**	**Time × Group effect**
Type I muscle strength	Control group	16.48 (1.87)	18.55 (2.51)	27.48 (1.87)	*F* = 27.02	*F* = 48.47	*F* = 207.02
	Intervention group	16.12 (2.25)	25.08 (2.26)^***^	29.22 (2.25)^***^	<0.0001	<0.0001	<0.0001
Type II muscle strength	Control group	22.82 (1.92)	23.98 (1.87)	31.27 (1.79)	*F* = 21.93	*F* = 82.08	*F* = 104.40
	Intervention group	22.60 (2.11)	29.32 (2.25)^***^	34.90 (2.06)^***^	<0.0001	<0.0001	<0.0001
Pelvic floor muscle tension	Control group	11.19 (2.25)	8.45 (1.96)	4.79 (2.25)	*F* = 28.14	*F* = 11.98	*F* = 152.70
	Intervention group	12.18 (2.09)^*^	5.71 (1.58)^***^	2.85 (0.89)^***^	<0.0001	0.0008	<0.0001

* indicated the comparison between groups within each time period, *P < 0.05 and

*** *P < 0.001*.

**Table 4:** Outcomes associated with sexual function.

**Outcomes**	**Groups**	**Before intervention**	**6 weeks**	**12 weeks**	**Time effect**	**Group effect**	**Time × Group effect**
FSFI	Control group	20.51 (2.33)	21.97 (2.38)	26.81 (1.66)	*F* = 7.38	*F* = 17.73	*F* = 128.66
	Intervention group	20.62 (2.57)	26.51 (2.02)^***^	26.97 (1.92)	0.0008	<0.0001	<0.0001
Sexual desire	Control group	2.69 (0.43)	3.07 (0.76)	4.73 (0.58)	*F* = 10.00	*F* = 27.35	*F* = 104.39
	Intervention group	2.60 (0.55)	4.57 (0.60)^***^	4.73 (0.61)	<0.0001	<0.0001	<0.0001
Arousal	Control group	2.65 (0.66)	2.87 (0.79)	4.78 (0.63)	*F* = 17.79	*F* = 22.90	*F* = 204.87
	Intervention group	2.57 (0.74)	4.64 (0.62)^***^	4.78 (0.59)	<0.0001	<0.0001	<0.0001
Damp	Control group	3.94 (0.72)	3.93 (0.73)	3.92 (0.73)	*F* = 1.18	*F* = 0.05	*F* = 0.45
	Intervention group	3.88 (0.70)	3.93 (0.74)	3.93 (0.76)	0.3095	0.8230	0.6384
Climax	Control group	3.36 (0.59)	3.65 (0.53)	4.11 (0.49)	*F* = 0.19	*F* = 2.18	*F* = 13.19
	Intervention group	3.34 (0.68)	4.08 (0.65)^***^	4.16 (0.57)	0.8307	0.1429	<0.0001
Pain	Control group	4.00 (0.55)	4.01 (0.55)	4.14 (0.43)	*F* = 1.18	*F* = 1.97	*F* = 1.17
	Intervention group	4.16 (0.35)	4.15 (0.38)	4.18 (0.40)	0.3098	0.1632	0.3122
Satisfaction	Control group	3.83 (0.76)	4.44 (0.56)	5.13 (0.41)	*F* = 0.23	*F* = 14.07	*F* = 11.27
	Intervention group	4.08 (0.89)	5.14 (0.49)^***^	5.19 (0.51)	0.7953	0.0003	<0.0001

* *indicated the comparison between groups within each time period*.

*** *P < 0.001*.

## Discussion

Bladder training includes lifestyle modification and use of relaxation and distraction techniques to control urinary frequency and urgency (23). It has been revealed that bladder training, including PFMT, is widely used in behavioral and physical therapies for OAB (24). Accumulating evidence has confirmed that PFMT can be combined with other treatment modalities to prevent OAB symptoms (25). However, a recent systematic review shows that the literature regarding the effectiveness of PFMT on improvement of OAB remains inconclusive and heterogeneous (26).

This study aimed to observe the efficacy of Yun-type optimized pelvic floor training therapy on the improvement of OAB and sexual function, to provide guidance for the treatment of severe OAB. By comparing the clinical efficacy of Yun-type optimized pelvic floor training combined with solifenacin vs. solifenacin alone for patients with severe OAB, we found that the OAB-associated outcomes (OABSS, urgent urination, urine, nocturia, UUI, PPBC, UDI-6, IIQ-7, VV, Qave, and Qmax), indicators related to PFM function (type I and type II muscle strength and PFM tension), and sexual function-associated indicators (FSFI, sexual desire, arousal, orgasm, and satisfaction) were significantly improved in both intervention groups. The improvements of these indicators in the intervention group were superior to the control group after 6 weeks of treatment. Moreover, improvements in OAB-associated outcomes (OABSS, nocturia, urgent urination, PPBC, VV, Qave, and Qmax), and PFM function-associated outcomes (type I and type II muscle strength and PFM tension) were also superior in the intervention group than the control group after 12/14 weeks of treatment. Other indicators did not exhibit significant differences between the two groups. These data merit further discussion.

Yun-type optimized pelvic floor training therapy combines professional and scientific PFMT with fashionable and sexy dance. Using this therapy, the groin, pelvic floor, perineum, waist, abdomen, chest, and arms can be exercised. The most prominent characteristic of this therapy is exercises involving the waist, abdomen, hip, and leg. Various groin and leg exercises can produce different weight training effects on PFM groups. In a previous study, dance training was found to significantly improve symptoms in patients with fibromyalgia, including reduced pain and improved functional capacity, QoL, and self-esteem (27). An et al. confirmed that belly dancing can improve the clinical symptoms of middle-aged women with urinary incontinence by increasing the vaginal pressure and strengthening the adductor muscle (28). Moreover, it is reported that an intervention involving pelvic muscle exercise can improve the symptoms of urinary incontinence by increasing the maximum pressure and duration of the PFM contraction (29). Although the efficacy of PFMT in patients with OAB has been studied, inconsistent results have been reported (25, 30). In this study, the following reasons might be responsible for the better clinical efficacy of Yun-type optimized pelvic floor training therapy for the improvement of OAB symptoms: (1) hip training could cause PFM contraction, thereby inhibiting excessive detrusor activity; (2) relaxation of the internal and external urethral sphincters could be inhibited by contraction of the PFM group, thus inhibiting urination; (3) this therapy could improve the associated mental disorders caused by pelvic floor dysfunction; and (4) groin training could increase groin flexibility and vaginal control, as well as further improve the FSFI.

The effectiveness of solifenacin in patients with OAB has already been well-illustrated. It has been reported that solifenacin was efficacious and well-tolerated in patients with OAB, and this treatment could improve QoL in patients (31). Nazir et al. demonstrated that the efficacy of solifenacin was similar to other antimuscarinics for OAB, but it had a lower risk of dry mouth (32). However, drug therapy alone does not completely relieve symptoms in most patients and is often accompanied by adverse effects. Increasing evidence has revealed that patients with OAB may benefit more from a combination of behavioral and drug therapy (33). Burgio et al. revealed that the combination of behavioral and drug therapies showed greater improvements in OAB symptoms than drug therapy alone and that it is reasonable to begin with behavioral therapy alone when using a stepped approach (34). The combination therapy may be more effective in improving frequency, urine output, incontinence and symptom distress (35, 36). In this study, patients in the intervention group experienced additional improvements in the OAB symptoms, PFM function, and sexual function compared to those in the control group. These results suggest that addition of Yun-type optimized pelvic floor training therapy to traditional drug therapies could better benefit women with severe OAB and SD. Moreover, the inclusion of younger women resulted in better compliance to the Yun-type optimized pelvic floor training therapy, which could improve confidence in women and subsequently yield benefits on SD.

Several limitations of this study should be mentioned: Firstly, the age range of patients was narrow, and we did not conduct stratified analyses based on patient characteristics owing to the small number of included patients. Secondly, the number of adverse events was lower than expected, but this was not recorded in this study. Thirdly, the follow-up time was short. Lastly, the analyses in this study were based on short-term interventions. More randomized controlled trials with larger sample sizes and long-term interventions should be performed to explore the clinical application of Yun-type optimized pelvic floor training for middle-aged women with severe OAB.

## Conclusion

In conclusion, our findings reveal that the use of Yun-type optimized pelvic floor training may yield additional benefits when combined with solifenacin for severe OAB and sexual function in middle-aged women. A combination of Yun-type optimized pelvic floor training with traditional drug therapies can improve clinical outcomes of severe OAB. This study will be helpful for the treatment of this disease.

## Data Availability Statement

The original contributions presented in the study are included in the article/Supplementary Material, further inquiries can be directed to the corresponding authors.

## Ethics Statement

Written informed consent was obtained from all participants, and the protocol was reviewed and approved by the Ethics Committee of the Shanghai Fifth People's Hospital Affiliated to Fudan University (No. 2017-041). The clinical trial registration was ChiCTR-INR-17012189.

## Author Contributions

YW, GS, and CS conceived and designed the research. CS, DZ, and YW participated in the acquisition of data. CS and WY analyzed and interpreted the data. YW, CS, and WY designed the study and performed the statistical analysis. GS participated in obtaining funding. CS, DZ, and WJ drafted the manuscript, conceived the study, and participated in its design and coordination. YW and GS revised the manuscript for important intellectual content. All authors contributed to the article and approved the submitted version.

## Conflict of Interest

The authors declare that the research was conducted in the absence of any commercial or financial relationships that could be construed as a potential conflict of interest.

## Abbreviations

OAB: overactive bladder
QoL: quality of life
UUI: urgency urinary incontinence
SD: sexual dysfunction
PFM: pelvic floor muscle
PFMT: pelvic floor muscle training
OABSS: overactive symptom bladder score
FSFI: female sexual function index
PVR: post-voiding residual
PPBC: patient's perception of bladder condition
UDI-6: urogenital distress inventory-6
IIQ-7: incontinence impact questionnaire-7
VV: voiding volume
Qave: average flow rate
Qmax: maximum flow rate
PVR: postvoid residual urine volume
SPSS: Statistical Package for the Social Sciences.
