# Supplementary figures and images for: Efficacy of Yun-Type Optimized Pelvic Floor Training Therapy for Middle-Aged Women With Severe Overactive Bladder: A Randomized Clinical Trial
Source: Front Surg. 2021 Jul 14;8:670123. doi: 10.3389/fsurg.2021.670123 (PMC8316598; doi:10.3389/fsurg.2021.670123)

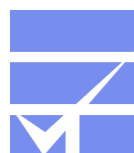

# CONSORT

TRANSPARENT REPORTING of TRIALS

## CONSORT 2010 Flow Diagram

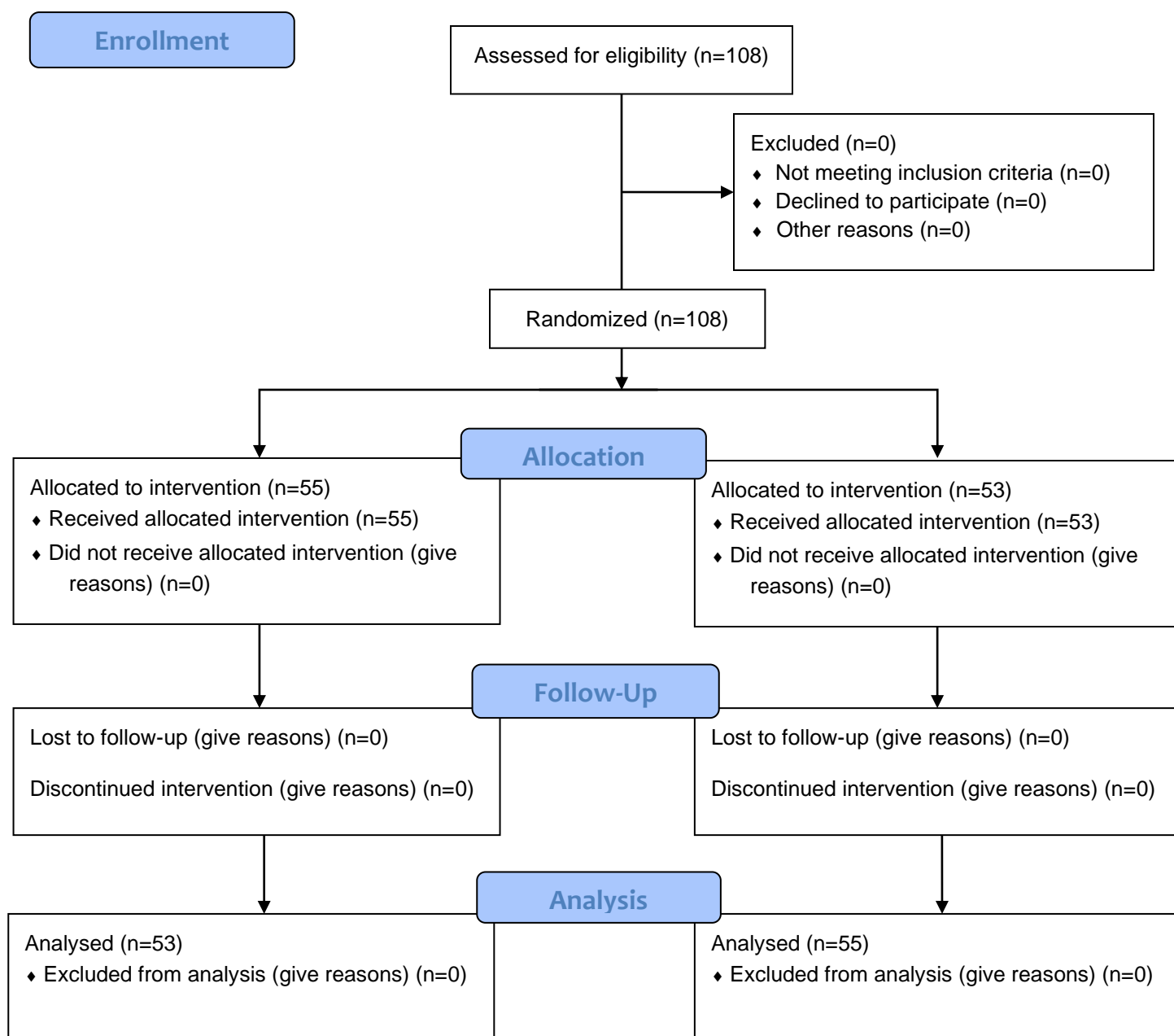

Supplement: Supplementary file 1 [file Data_Sheet_1.PDF]
